# Nuclei-level prior knowledge constrained multiple instance learning for breast histopathology whole slide image classification

**DOI:** 10.1016/j.isci.2024.109826

**Published:** 2024-04-26

**Authors:** Xunping Wang, Wei Yuan

**Affiliations:** 1 School of Remote Sensing and Information Engineering, Wuhan University, 129 Luoyu Road, Wuhan 430079, China; 2 Co-Creation Center for Disaster Resilience, International Research Institute of Disaster Science, Tohoku University, Aoba 468-1, Aramaki, Aoba-ku, Sendai 980-8572, Japan

**Keywords:** Bioinformatics, Machine learning

## Abstract

New breast cancer cases have surpassed lung cancer, becoming the world’s most prevalent cancer. Despite advancing medical image analysis, deep learning’s lack of interpretability limits its adoption among pathologists. Hence, a nuclei-level prior knowledge constrained multiple instance learning (MIL) (NPKC-MIL) for breast whole slide image (WSI) classification is proposed. NPKC-MIL primarily involves the following steps: Initially, employing the transfer learning to extract patch-level features and aggregate them into slide-level features through attention pooling. Subsequently, abstract the extracted nuclei as nodes, establish nucleus topology using the K-NN (K-Nearest Neighbors, K-NN) algorithm, and create handcrafted features for nodes. Finally, combine patch-level deep learning features with nuclei-level handcrafted features to fine-tune classification results generated by slide-level deep learning features. The experimental results demonstrate that NPKC-MIL outperforms current comparable deep learning models. NPKC-MIL expands the analytical dimension of WSI classification tasks and integrates prior knowledge into deep learning models to improve interpretability.

## Highlights

• A multiscale multiple instance learning model for whole slide image classification
• Integrate prior knowledge into deep learning models to improve interpretability
• Expand the analytical dimension of existing whole slide image classification algorithms by introducing nuclei-level features

## Graphical abstract

**Figure:**
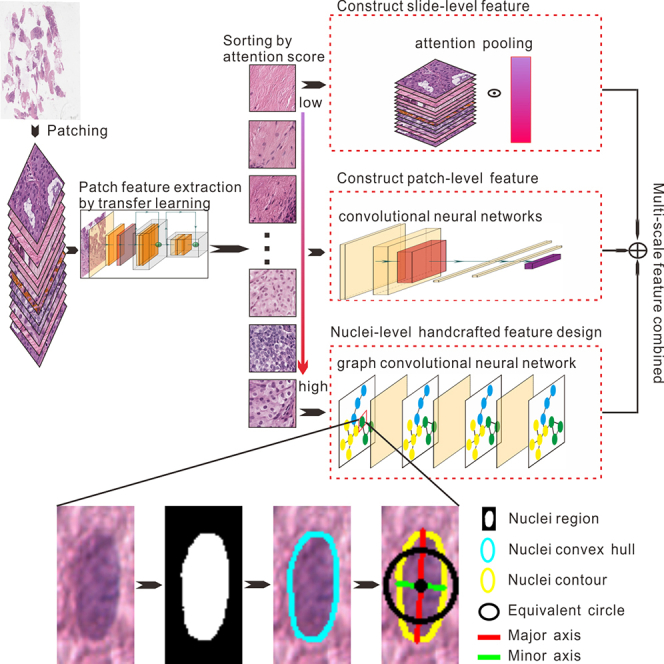

## Introduction

In recent years, the incidence of new breast cancer cases has surged rapidly, surpassing lung cancer to become the world’s most prevalent cancer type.^1^ With the advancement of histological section digitization technology, whole slide image (WSI) offers the advantage of presenting tissue morphology with exceptionally high resolution.^2^ Also, the characteristic of extremely high resolution in WSI makes it possible to classify breast cancer,^3^ segment lesion areas,^4^ analyze the microenvironment,^5^ and assess prognosis.^6^

The conventional classification process for pathological images adheres to the typical image classification steps: target segmentation, feature extraction, feature selection, and disease diagnosis. These four steps are carried out independently and then integrated for tuning. However, errors are inevitable in each step, and their accumulation can result in unreliable classification outcomes. Additionally, most traditional machine learning algorithms require loading all data into memory at once, which is not feasible for WSIs with gigapixels.^7^

The classification process based on deep learning is end to end.^8^ Thanks to the weight-sharing strategy^9^ and the rapid advancement in computer computing power,^10^ deep learning methods have made significant strides in the field of computer vision, propelling advancements in medical image analysis.^11^

Given the gigantic size of WSI, such as 100,000 × 100,000 pixels, the analysis scale of WSI progresses through two stages, from patch level to slide level.

Conventional deep learning methods cannot be directly applied for classifying WSI^12^ and must be processed in blocks.^13^^,^^14^^,^^15^ Although processing in blocks reduces the algorithm’s demands on computer hardware, it disrupts the integrity of the organization and compromises spatial information. The analysis of the entire WSI should fully consider spatial context information rather than being based on arbitrary patches.^16^ Therefore, most current studies aggregate patch-level features to the slide-level before making inferences.^17^^,^^18^^,^^19^

At present, the improvement of the deep learning algorithm based on MIL focuses on how to model the spatial relationship of patches to better aggregate patch-level features into slide-level features, while ignoring the influence of nuclear-level features on the final classification results. The distribution and morphology of nuclei are an essential basis for cancer diagnosis.^20^ Although deep neural networks can theoretically approximate any function model,^21^ it is still not widely recognized by pathologists due to its lack of interpretability.^22^

To fully leverage the capacity of deep learning methods in model fitting, while also considering the incorporation of handcrafted features with clear interpretations to enhance the interpretability of the model, this paper intends to investigate a way for classifying WSI constrained by nuclear-level handcrafted features.

The rest of the paper is arranged as follows: A brief review of some related work is presented below. Detailed ablation experiments and comparison studies are presented in the results section, followed by the discussion and conclusion part in the discussion section. Finally, a detailed overview of the methodology is provided in the STAR methods.

The combination of extremely large image sizes and the high expertise threshold in pathology makes it prohibitively expensive to acquire pixel-level WSI labeling data. However, acquiring slide-level labeling is comparatively more accessible. The scenario of 'The class of the entire WSI is known, while the class of all patches that make up the WSI is unknown' aligns precisely with the application of Multiple Instance Learning. In MIL, the training set comprises bags with known class labels, where each bag includes instances with unknown labels. A bag is labeled as positive if it contains at least one positive instance, and if all instances in a bag are negative, the bag is marked as negative.^23^

Hence, an increasing number of researchers have approached digital pathological image classification as a weakly supervised classification problem based on the MIL algorithm. Within MIL-based deep learning algorithms, one of the most intuitive ideas is to aggregate patch-level features extracted by Convolutional Neural Network (CNN) into slide-level features through pooling operations.^24^ Ilse et al.^25^ take ‘the fundamental theorem of symmetric function and the fact that neural network layer has arrangement equivariant’^26^ as the principle for network design. They propose an attention-based deep MIL algorithm, utilizing the attention mechanism to assign more flexible weights to patches. Li et al.^27^ innovatively incorporated a non-local attention mechanism to tackle the MIL problem. Through evaluating the similarity between the patch with the highest score and other patches, distinct attention weights were assigned to each patch, thereby optimizing the aggregation of patch-level features into comprehensive slide-level features. This approach aligns with analogous studies reported in existing literature.^28^^,^^29^^,^^30^

Nevertheless, the aforementioned studies are contingent upon the assumption that all instances in each bag are independent and equally distributed. In reality, instances often exhibit correlations,^31^ necessitating the modeling of inter-instance relationships before applying MIL. Shao et al.^32^ challenge the assumption of independent distribution, proposing a scoring function based on Hausdorff distance to assess correlations between instances. They leverage the Transformer framework^33^ to incorporate the spatial topological relationships among similar patches. Philip et al.^34^ select the c instances with the highest score using the instance classifier. Subsequently, they calibrate the distribution of instances by subtracting the features of these top-scoring c samples from the features of all instances in the bag. However, operating under the assumption that 'cancerous WSI have more information than normal WSI,' their model tends to prioritize features of cancerous WSI, thereby attenuating the recognition of features in normal WSI.

As the bag contains numerous instances (tens of thousands), refining the instances is necessary to reduce the number involved in calculations. Xie et al.^35^ adopted the feature clustering method to divide the instances in the bag into e categories and then the characteristics of the instance where the centroid of e categories are fused as the characterization of the bag to make the final prediction. Lu et al.^36^ sorted the instances according to the level of attention scores, and then took the c instances with the highest and lowest attention scores as inputs to train a patch-level classifier to constrain the slide-level diagnosis results, and achieved better results than diagnosis based on slide-level features alone.

In addition, to enrich the diversity of training samples, it is necessary to explore the impact of different data enhancement methods on classification accuracy: Yang et al.^37^ proposed a “mix-the-bag” data enhancement method, which means adding, replacing, and interpolating among the instances of the bag of the same category to generate new virtual bags. Zhang et al.^38^ randomly select instances from the parent bag to form sub-bags and assign the label of the parent bag to sub-bags. However, sub-bags derived from positive parent bag may not necessarily contain positive instances, leading to the introduction of sub-bags with incorrect labels. To address this issue, they designed a feature distillation branch to eliminate errors.

To fully harness the potential of deep learning methods in model fitting and incorporate handcrafted features for building interpretable deep learning models, this paper aims to explore a method for classifying WSI constrained by nuclear-level handcrafted features.

## Results

### Construction of the NPKC-MIL

**Figure 1:**
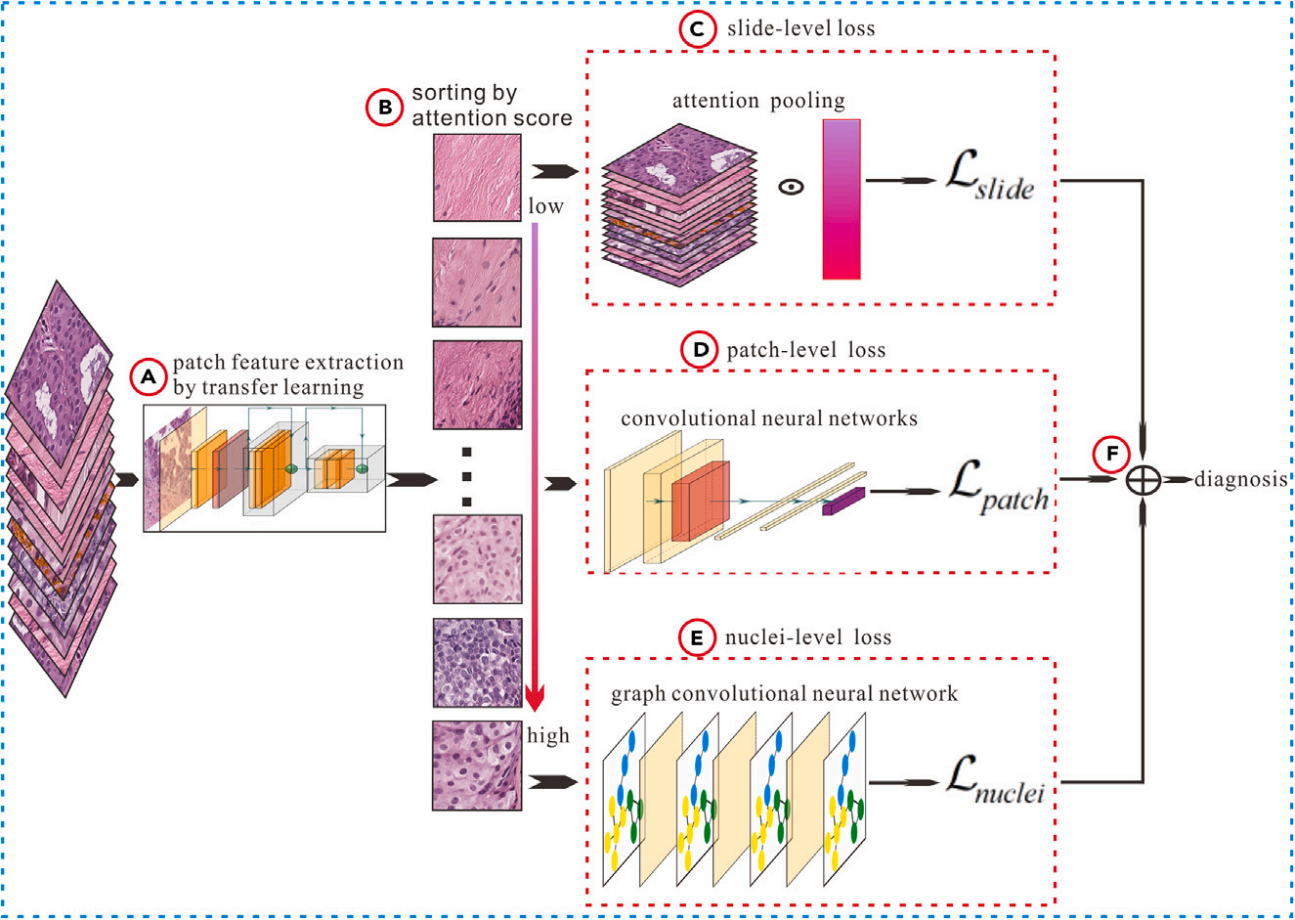
Flowchart of NPKC-MIL (A) Extracting patch feature by transfer learning. (B) Ranking the importance of patches by attention score. (C) Calculating slide-level loss. (D) Calculating patch-level loss. (F) Constructing total loss function.

The construction process of nuclei-level prior knowledge constrained multiple instance learning (NPKC-MIL) can be summarized as follows: Initially, patch-level features are extracted from WSI and sorted based on the importance of patches. Subsequently, nucleus features (treat extracted nuclei as Graph nodes) are aggregated and updated using the Graph Convolutional Network (GCN). The losses are then calculated at the slide-level, patch-level, and nuclei-level. Finally, patch-level and nuclei-level losses are considered as penalty items and added to the total loss function for model training, as depicted in Figure 1.

Figure 1 outlines the four steps involved in constructing NPKC-MIL: First, utilizing the transfer learning method to extract patch-level features (Figure 1A) and rank the importance of patches using an attention mechanism (Figure 1B). Second, aggregating patch-level features into slide-level features through attention pooling and calculating patch-level loss $L_{patch}$ (Figure 1C) and slide-level loss $L_{slide}$ (Figure 1D) separately. Third, abstracting the extracted nuclei as nodes, constructing the node topology using the K-NN algorithm, extracting nuclei-level handcrafted features, aggregating and updating node information with GCN, and calculating nuclei-level loss $L_{nuclei}$ (Figure 1E). Fourth, considering $L_{patch}$ and $L_{nuclei}$ as penalty terms, they are added to the total loss function for model training (Figure 1F).

### Nuclei-level feature design

There exists a perceptual understanding that 'cancerous nuclei are larger, rounder, and messier than normal nuclei' in the clinical diagnosis process. To quantitatively articulate these differences and aid in handcrafted feature design, we conducted five randomized trials on selected experimental datasets. Each experiment involved the collection of 100,000 nuclei from both cancerous WSI and normal WSI for statistical analysis.

**Figure 2:**
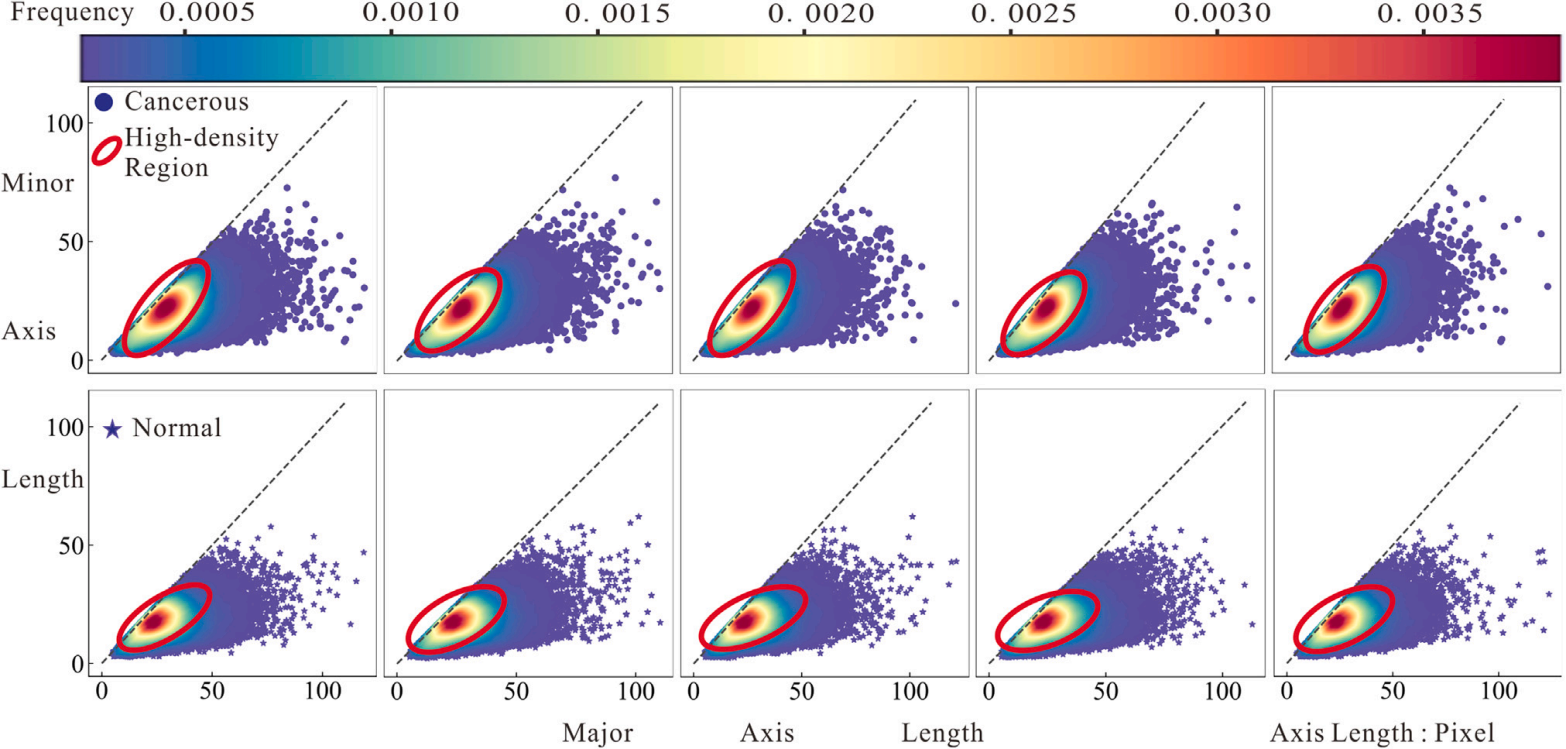
Nuclei scatter density map

Figure 2 illustrates the scatter density of normal and cancerous nuclei. A comparison of the high-density region in Figure 2 distinctly reveals that cancerous nuclei exhibit larger areas and a rounder shape.

**Figure 3:**
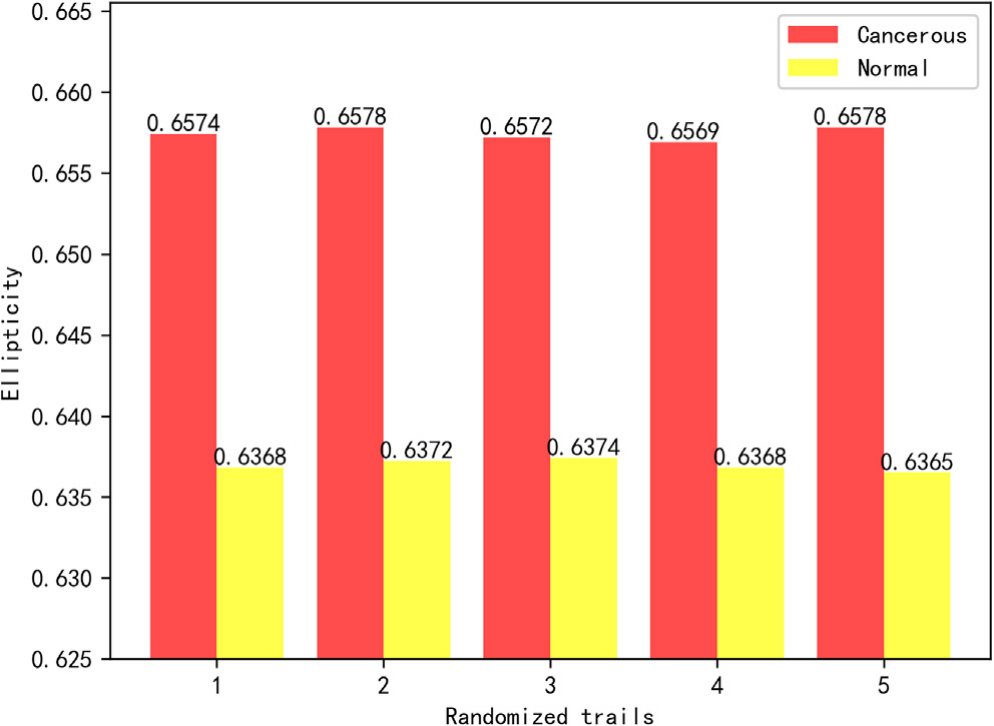
Mean nuclei ellipticity of five randomized trials

**Figure 4:**
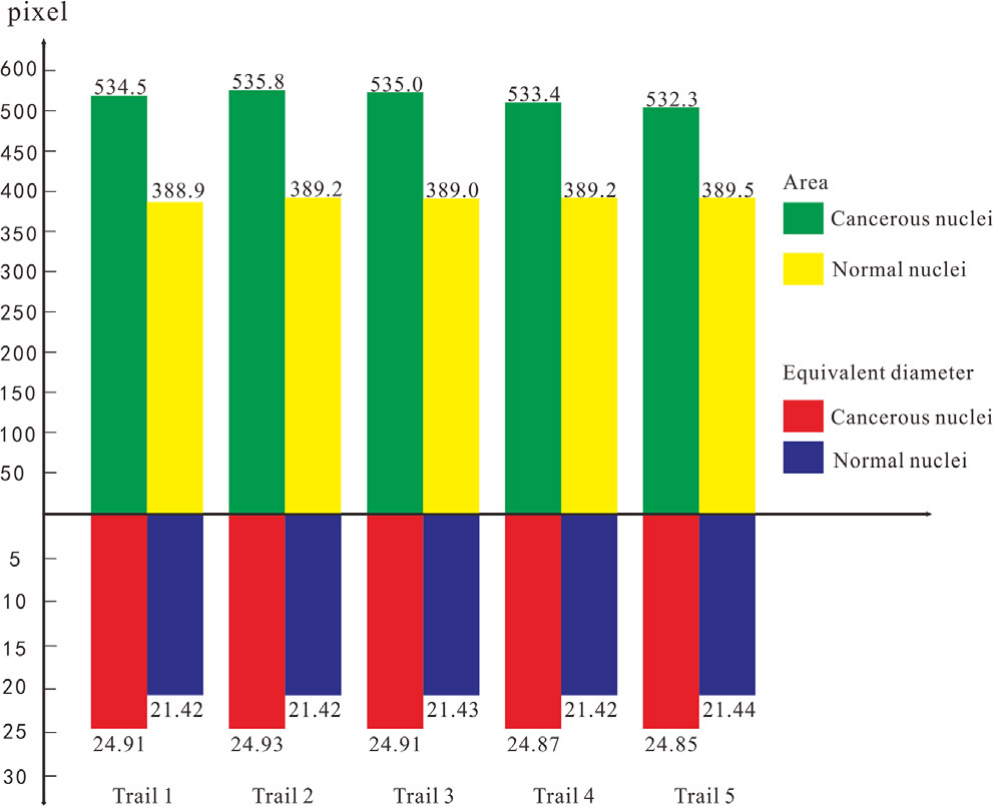
Area and equivalent diameter of normal nuclei and cancerous ones

The elliptical characteristic in Figure 3 suggests that cancerous nuclei are rounder. Additionally, the quantitative analysis of characteristic parameters such as area and equivalent diameter ellipticity in Figure 4 indicates that cancerous nuclei are larger.

#### Geometric features

**Figure 5:**
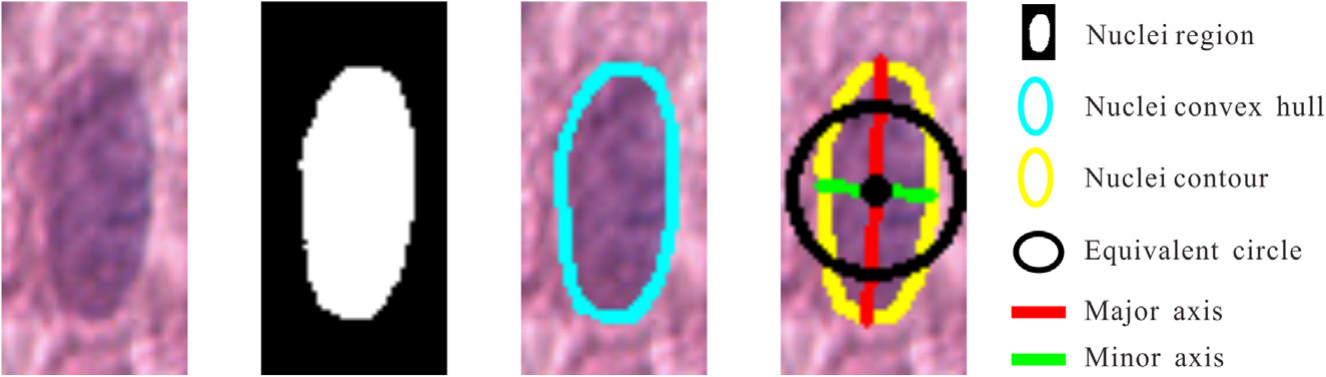
Schematic diagram of the geometric features of the nuclei in part

Hence, we extracted a set of 9-dimensional geometric features from the nucleus. These features encompass major axis length (*Major*), minor axis length (*Minor*), area (*Area*), direction *θ* (the angle between the major axis and horizontal direction), eccentricity (*Ecc*), ellipticity (*Ell*), equivalent diameter (*Dia*, the diameter of a circle with an area equal to the nuclei area), perimeter(*Per*), and convex hull area (*AreaHull*). Figure 5 shows geometric features in part.

#### Texture features

**Figure 6:**
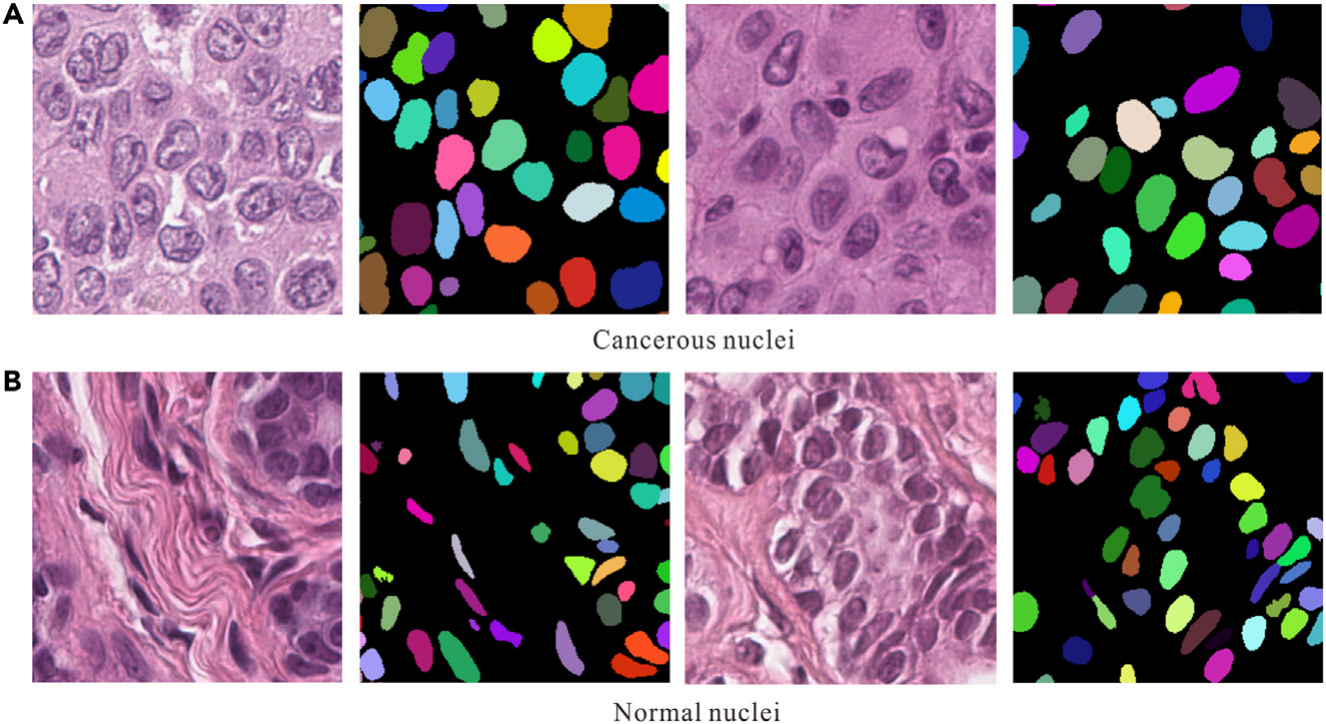
Nuclei texture (the different colors in the black background represent different nuclei) (A) Cancerous nuclei. (B) Normal nuclei.

Based on our understanding, cancerous nuclei exhibit a complex texture, whereas normal nuclei appear smooth. This observation is further supported by Figure 6, which illustrates the complexity of cancerous nuclei texture in contrast to the smooth texture of normal nuclei.

**Figure 7:**
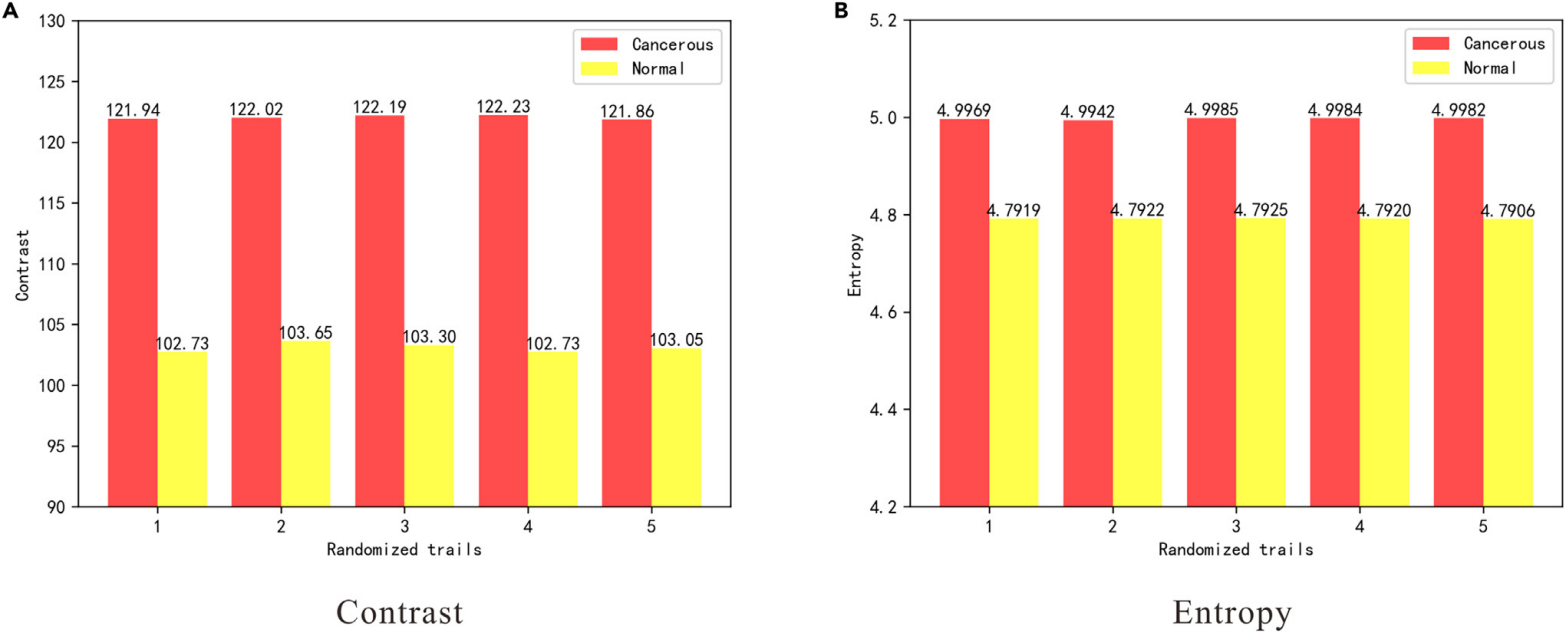
Mean nuclei contrast and entropy of five randomized trials (A) The contrast difference. (B) The entropy difference.

For a quantitative portrayal of the difference, Figure 7 illustrates two features characterizing nucleus texture. Comparative analysis reveals the following insights: 1) Cancerous nuclei exhibit more precise imaging, as indicated by the contrast characteristic reflecting the clarity of nuclei. This aligns with the clinical observation that cancerous nuclei appear hyperchromatic during H&E staining; 2) The texture of cancerous nuclei is more intricate, as evidenced by the entropy characteristic. Entropy serves as a measure of the information content within the image, and a higher value indicates a more complex texture.

To this end, we extracted 7-dimensional texture features from the nuclei. These features encompass contrast (*Con*), dissimilarity (*Diss*), homogeneity (*Hom*), entropy (*Ent*), angular second movement (*ASM*), roughness (*Rou*), and dispersion (*Dsip*).

To visually illustrate the distinction between the corresponding characteristics of cancerous nuclei and normal nuclei, Table 1 presents the ratio of 16-dimensional features in 5 randomized trials. The color characteristics are excluded due to the substantial H&E color difference, as evident in Figure 6. A more detailed modeling process is described in the STAR methods section.

**Table 1:** Ratio of handcrafted features between cancerous nuclei and normal ones

Handcrafted features		Mean	STDEV
Geometric Features	Area	1.3726	1.4885
	Convex hull area	1.3664	1.4497
	Eccentricity	0.9751	1.0190
	Equivalent diameter	1.1620	1.2875
	Major axis length	1.1302	1.0945
	Minor axis length	1.1913	1.3453
	Direction	1.4527	0.9916
	Perimeter	1.1607	1.1888
	Ellipticity	1.0321	0.9849
Texture Features	Roughness	0.9962	1.0893
	Contrast	1.1839	0.9701
	Dissimilarity	1.1366	0.9712
	Homogeneity	0.8425	0.8064
	Entropy	1.0429	1.0463
	Angular second moment	0.7735	0.9690
	Dispersion	1.1284	1.0542

### Dataset description

Currently, prominent public datasets relevant to auxiliary breast cancer diagnosis include BACH2018,^39^ BreakHis,^40^ and BRACS.^41^ The BACH2018 dataset comprises 10 normal WSIs and 20 cancerous WSIs. BRACS stands out as the most extensive dataset available to the author, with 547 WSIs post-update; however, only 423 WSIs were effectively obtained during this paper’s experimentation. Within these, 252 were normal, and 171 were cancerous, resulting in a positive/negative sample ratio of 40.43%:59.57%. BreakHis, offering only patches cropped from WSIs without enabling the construction of slide-level features, is not utilized in this study. The provided data includes labeling information, with '0' denoting normal WSIs and '1' indicating cancerous WSIs.

WSIs exhibit strong heterogeneity and diversity stemming from various sources such as different image acquisition devices, scanning protocols, image gatherers, variations in the H&E staining process before scanning, and inherent differences among patients. These factors contribute to distribution drift in WSI images, and the diverse set of samples significantly influences the model’s generalization ability. A model trained on an imbalanced sample set may acquire biased information about sample proportions, leading to predictions that overly focus on certain categories and compromise the model’s robustness.^42^

To enhance sample diversity and maintain a balanced ratio of positive and negative samples, this study incorporates 20 cancerous WSIs from the BACH2018 dataset and 33 self-owned cancerous WSIs in addition to the BRACS dataset. The experimental dataset comprises 476 WSIs with a positive/negative sample ratio of 47.06%:52.94%, as depicted in Table 2.

**Table 2:** Construction of experimental datasets

Image source	Collecting Device	Image Format	Pixel Size (μm)	Normal WSIs	Cancerous WSIs
BRACS	Aperio AT2	.svs	0.25 × 0.25	252	171
BACH2018	Leica SCN400	.svs	0.42 × 0.42	0	20
Self-owned data	KF-PRO-005-EX	.kfb	0.50 × 0.50	0	33
Total				252	224

### Implementation details

**Figure 8:**
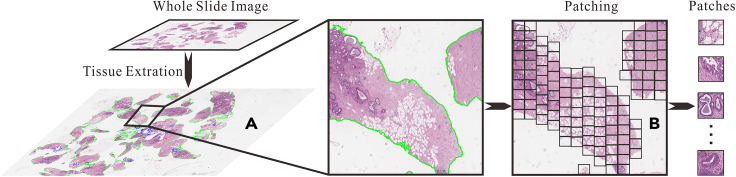
Tissue extraction and patching (the green line indicates the tissue contour, and the small rectangular box in step B are sliding window of 512×512 pixels to extract patches for training) (A) Extracting tissue. (B) Slicing WSI into patches.

Typically, human tissue does not cover the entire slide during slide preparation, resulting in WSIs containing significant blank spaces, as depicted in Figure 8. Efficiently extracting the human tissue region is crucial for conserving computing resources and enhancing the algorithm’s efficiency.

The process begins by leveraging the principle that the S-channel of the HSV color model represents a combination of specific spectral colors and white.^43^ WSIs are then transformed from RGB to HSV in the high pyramid (e.g., layer 4). Subsequently, the human tissue region is extracted using the OSTU method^44^ in the S-channel, and any small holes in the tissue are filled through mathematical morphology closing operations.^45^ The extracted human tissue contour is filtered based on region thresholds, and the silhouettes are restored to the original image layer.

Furthermore, it’s impracticable to load an entire WSI into the GPU directly, due to its large size (e.g., 100,000 × 100,000 pixels), so a patch-based processing approach should be employed.^46^ This involves using a sliding window of 512 × 512 pixels on the WSI within the extracted human tissue area, with the top-left coordinates of each sliding window recorded. The final step involves restoring the WSI based on these coordinates. Figure 8 illustrates the step-by-step processing procedure.

According to the percentage of 7:1:2, the experimental images were divided into training datasets, verification datasets, and test datasets. All experiments were performed on a single desktop computer with Window10 operating system, CPU with six cores and six threads, 3.00 GHz i5-8500 (32 GB memory), and the GPU GTX2080 with 8 GB memory.

### Ablation experiment

To validate the effectiveness of handcrafted feature constraints, three sets of ablation experiments were conducted. The classification result based on slide-level features is denoted as ablation1 (macro feature), patch-level deep learning feature constraints are characterized as ablation2 (meso feature), and nuclei-level handcrafted feature constraints are indicated as ablation3 (micro feature). For simplicity, the model inference probability is denoted as PrePro (Predicted Probability).

As shown in Table 3, for WSIs (WSI-1, WSI-2) characterized by clear imaging and distinct features, both NPKC-MIL and the ablation experiments consistently achieve accurate classification with high probability.

**Table 3:** Performance of different ablation experiments on four test samples

Methods	WSI-1(GT = 0)		WSI-2(GT = 1)		WSI-3(GT = 0)		WSI-4(GT = 1)	
	PrePro	PreCls	PrePro	PreCls	PrePro	PreCls	PrePro	PreCls
NPKC-MIL	0.9249	0	0.7999	1	0.5753	0	0.5220	1
ablation1	0.9996	0	0.9998	1	0.9988	1	0.9267	0
ablation2	0.9997	0	0.9998	1	0.6992	1	0.6487	0
ablation3	0.8872	0	0.6998	1	0.8867	1	0.9487	0

PrePro is an abbreviation for “predict probability”, PreCls is an abbreviation for “predict class”.

**Figure 9:**
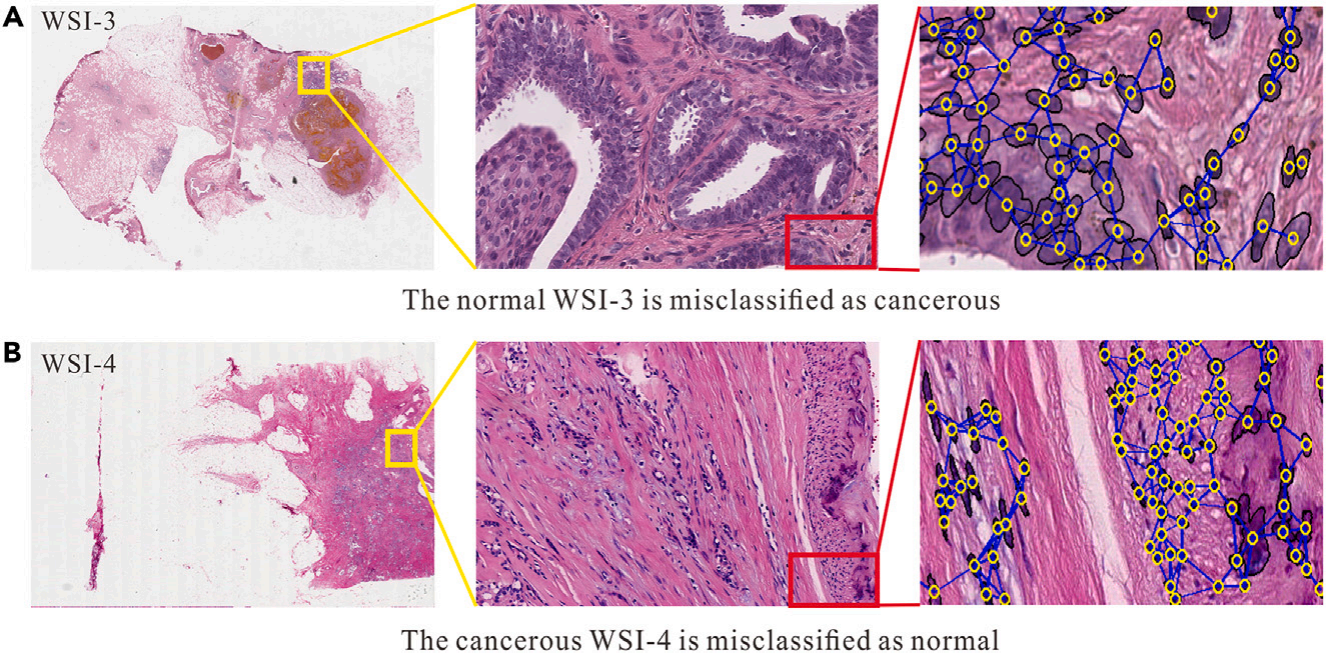
The misclassified test samples and their macro, meso, and micro characteristics diagram (the yellow circle is the nuclei centroid, the black circle is the nuclei contour, and the blue lines represent the adjacency relationship of nuclei)

For WSI-3, with a subclass as Flat Epithelial Atypia (FEA), which appears cancerous at the macro view (Figure 9A), ablation1 misclassified it as cancerous with a very high probability (0.9988). However, ablation2 significantly reduced the misclassification probability to 0.6992 by introducing the 1024-dimensional meso feature constraint. Although ablation3 is constrained by 16-dimensional micro features, its misclassification probability remains high (0.8867). This suggests that micro features play a restrictive role, but their impact is not as pronounced as that of the 1024-dimensional meso features, underscoring that the latter provides more effective constraint information than the former.

For WSI-4, with a subclass as invasive carcinoma (IC), ablation1 misclassified it as normal based on macro features (Figure 9B). However, upon introducing meso feature constraints (ablation2), the misclassification probability significantly decreased to 0.6487, indicating the effectiveness of the 1024-dimensional meso feature constraints. In contrast, relying solely on the micro 16-dimensional handcrafted feature constraint led to a high probability of misclassification as normal (0.9487). This emphasizes the challenge of exclusively focusing on micro features, encountering the dilemma of 'seeing the trees but not the forest.' Therefore, a combination of meso and micro characteristics is essential to constrain macro features correctly. Notably, NPKC-MIL classified it as cancerous with a probability of 0.5220.

**Figure 10:**
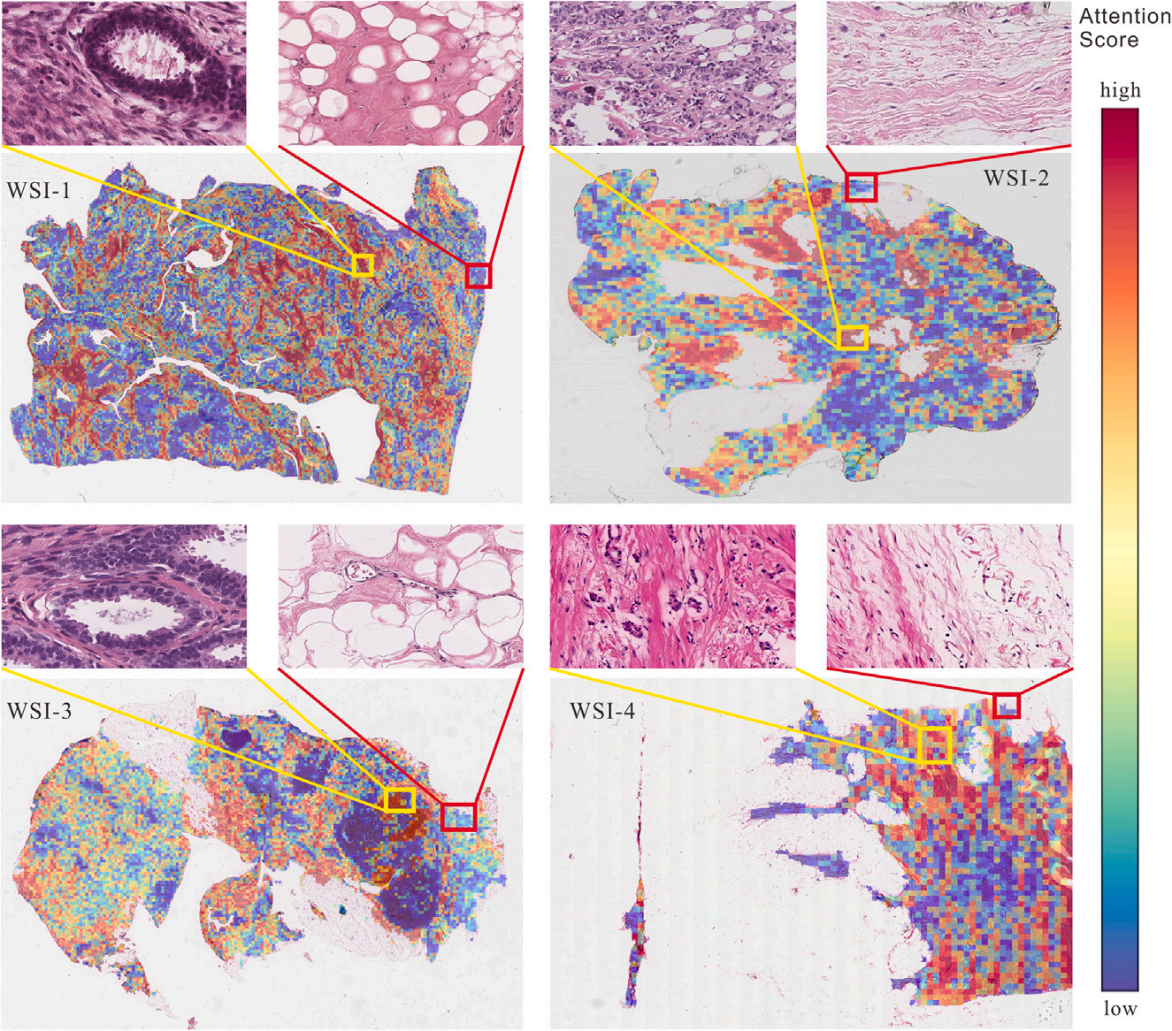
Attention heatmaps of the four test samples (yellow boxes for high attention score areas, red boxes for low attention score areas)

The attention score heatmap in Figure 10 reveals that regions with high attention predominantly consist of nuclei-rich areas, while regions with low attention are primarily composed of muscle or fat tissue. Hence, a more comprehensive analysis at the nuclear level is crucial for the areas exhibiting high attention.

Ultimately, as demonstrated in Table 4, the precision and accuracy of ablation2 and ablation3 surpass those of ablation1, underscoring the effectiveness of patch-level or nuclei-level characteristics in constraining slide-level features. However, the sensitivity (SE) of ablation3 is lower than that of ablation1, indicating that the detection of WSI carcinomatosis solely through handcrafted restrictions at the nuclear level is insufficient.

**Table 4:** Comparison table of ablation experiments

	AC(%)	SP(%)	SE(%)	PC(%)
NPKC-MIL	96.25	97.50	95.00	97.44
ablation1	83.75	85.00	82.50	84.62
ablation2	86.25	90.00	82.50	89.19
ablation3	85.00	90.00	80.00	88.89

Ablation2 outperforms ablation3 in terms of accuracy (AC), sensitivity (SE), and precision (PC), suggesting that patch-level deep learning features (1024 dimensions) offer more constraint information than nuclei handcrafted features (16 dimensions), albeit with marginal improvement. This analysis also fully illustrates that although deep learning methods can extract features of any dimension theoretically, the dimension of features is not the higher, the better, but rather to extract features with discriminative power. Finally, the amalgamation of nuclei-level and patch-level features enhance the constraint on slide-level features, thereby improving the accuracy of cancerous WSI classification.

### Comparative analysis of different methods

The performance of various comparison methods on four test samples is presented in Table 5. For WSIs with clear imaging and distinct features (WSI-1, WSI-2), except for TransMIL’s misclassification of WSI-1 as cancerous, other methods correctly classify WSI-1 with high probability.

**Table 5:** The performance of different comparison methods on four test samples

Methods	WSI-1(GT = 0)		WSI-2(GT = 1)		WSI-3(GT = 0)		WSI-4(GT = 1)	
	PrePro	PreCls	PrePro	PreCls	PrePro	PreCls	PrePro	PreCls
NPKC-MIL	0.9249	0	0.7999	1	0.5753	0	0.5220	1
TransMIL	0.6714	1	0.9963	1	0.9095	1	0.9662	1
FRMIL	0.5877	0	0.8821	1	0.8752	1	0.6288	0
CLAM-SB	0.9999	0	1.0000	1	0.9916	1	1.0000	0
ReMix	0.9914	0	1.0000	1	0.9466	0	0.9799	0
DTFD-MIL	1.0000	0	1.0000	1	0.9999	1	0.9022	1

PrePro is an abbreviation for “predict probability”, PreCls is an abbreviation for “predict class”.

**Figure 11:**
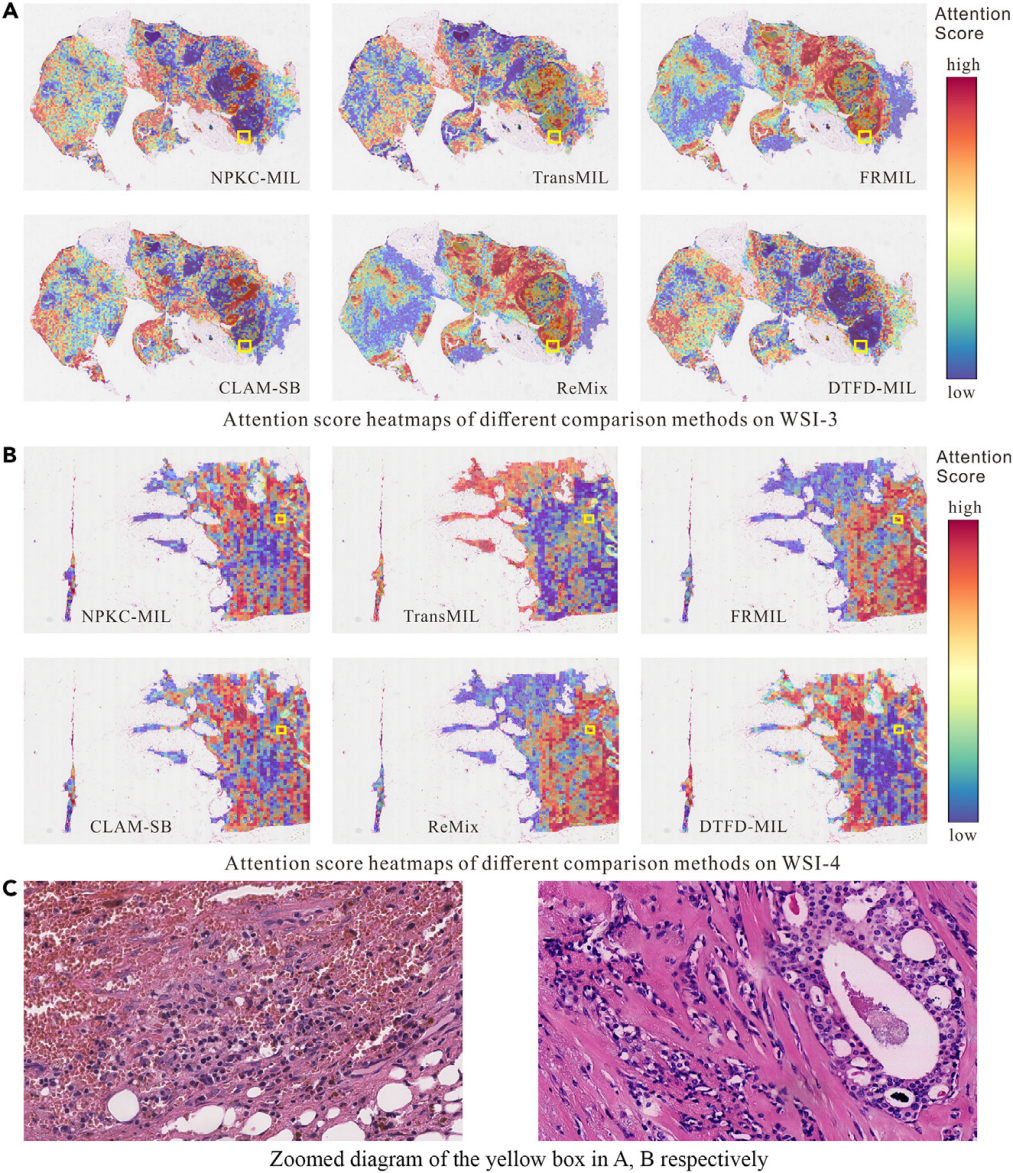
Attention score heatmaps of different comparison methods on WSI-3 and WSI-4 (yellow boxes are areas of high attention score) (A) Attention score heatmaps of different methods on WSI-3. (B) Attention score heatmaps of different methods on WSI-4. (C) Zoomed diagram of the yellow box in (A) and (B).

Figure 11C reveals that areas with high attention scores exhibit a complex texture comprising red blood cells, muscle cells, lymphocytes, epithelial cells, etc. Aside from DTFD-MIL, other methods assign higher attention to these areas, and the attention heatmap of FRMIL and ReMix is identical, indicating the shared use of the same attention framework.

NPKC-MIL accurately classifies WSI-3 and WSI-4. TransMIL misclassifies WSI-3 as normal but correctly classifies WSI-4. FRMIL incorrectly classifies WSI-3 as cancerous WSI, aligning with its assumption that “cancerous WSIs have more information than normal ones.” CLAM-SB misclassifies WSI-3 and WSI-4, suggesting that for WSIs with complex textures, meso features alone may not sufficiently constrain classification results based on macro features. In comparison to NPKC-MIL, ReMix correctly classifies WSI-3 with a higher probability (0.9466), and DTFD-MIL accurately classifies WSI-4 with a higher probability (0.9022), highlighting the simplicity and effectiveness of data enhancement for small sample data.

Table 6 presents a comparative analysis between NPKC-MIL and other five state-of-the-art methods.^29^^,^^31^^,^^33^^,^^34^^,^^35^ The results highlight that TransMIL, by treating patches as relevant entities and considering their spatial topological information through the Transfer framework, achieves an accuracy of 88.75% and a sensitivity of 90.00%. While FRMIL successfully adjusted the distribution of instances, its underlying assumption that “cancerous WSIs contain more information than normal ones” led the model to prioritize the features of cancerous WSIs, consequently diminishing its ability to recognize features in normal WSIs. This bias resulted in a lower specificity (67.5%) and a higher sensitivity (85%). Within the MIL framework, CLAM-SB employs a clustering concept and adds patch-level constraints with a few representative instances, achieving a sensitivity of 90.00%.

**Table 6:** Comparison of evaluation criteria of different algorithms

	AC(%)	SP (%)	SE (%)	PC (%)
NPKC-MIL	96.25	97.50	95.00	97.44
TransMIL^29^	88.75	87.50	90.00	87.80
FRMIL^31^	76.25	67.50	85.00	72.34
CLAM-SB^33^	86.25	82.50	90.00	83.72
ReMix^34^	86.25	92.50	80.00	91.43
DTFD-MIL^35^	83.75	87.50	80.00	86.49

The generalization ability of deep learning models is significantly influenced by the diversity of training samples. ReMix proposed the “mix-the-bag” data enhancement method, which is simple but effective, achieving an accuracy of 91.43%. DTFD-MIL uses pseudo-bags technology for data enhancement, but the child bags derived from the positive parent bags do not always contain positive instances, in which case will introduce a bag with a wrong label, and the feature distillation branch structure used in their study cannot eliminate the artificially introduced errors well, leading to a low SE with 80%.

It is noteworthy that all the aforementioned methods rely on slide-level and patch-level deep learning features from transfer learning models, neglecting micro-level prior knowledge (handcrafted features). In contrast, NPKC-MIL maximizes the potential of deep learning in feature extraction while incorporating meaningful manual features. This approach not only builds interpretable deep learning models but also achieves higher accuracy, specificity, and sensitivity, respectively.

## Discussion

### How do advancements in AI technologies drive the progress of digital histopathology research?

The image classification process involves a mathematical exploration to identify the mapping relationship between image features and target types. Traditional methods, rooted in feature engineering, rely on effective and comprehensive handcrafted features. In simpler scenarios, the objective function can be determined through sparse regression, and parameters can be solved using least squares.

However, for the challenging task of WSI classification, low-level handcrafted features (including color features, texture features, morphological features, etc.) prove insufficient in capturing the complexity of medical images. As illustrated in Table 4, the impact of 16-dimensional handcrafted feature constraints is inferior to that of deep learning features, underscoring the richer constraint information provided by deep learning features. Additionally, for WSIs exhibiting strong heterogeneity, modeling complex scenes through symbolic mathematical methods poses challenges due to the optimization space’s intricate structures and coefficients, making it arduous for optimization algorithms, such as simulated annealing and genetic algorithms, to find solutions. Lastly, many traditional feature-based engineering algorithms necessitate loading all data into memory simultaneously, which is impractical for WSIs with gigapixels.

### Why should we pay more attention to prior knowledge at the nucleus level?

Different from parametric models that can be explicitly expressed through mathematical formulas, deep learning operates as a statistical modeling method with unknown structures and coefficients in its equations. It is a data-driven approach optimized by gradient descent algorithms. While the convolution operation can theoretically extract features of any dimension, the lack of interpretability has hindered widespread acceptance among pathologists.

**Figure 12:**
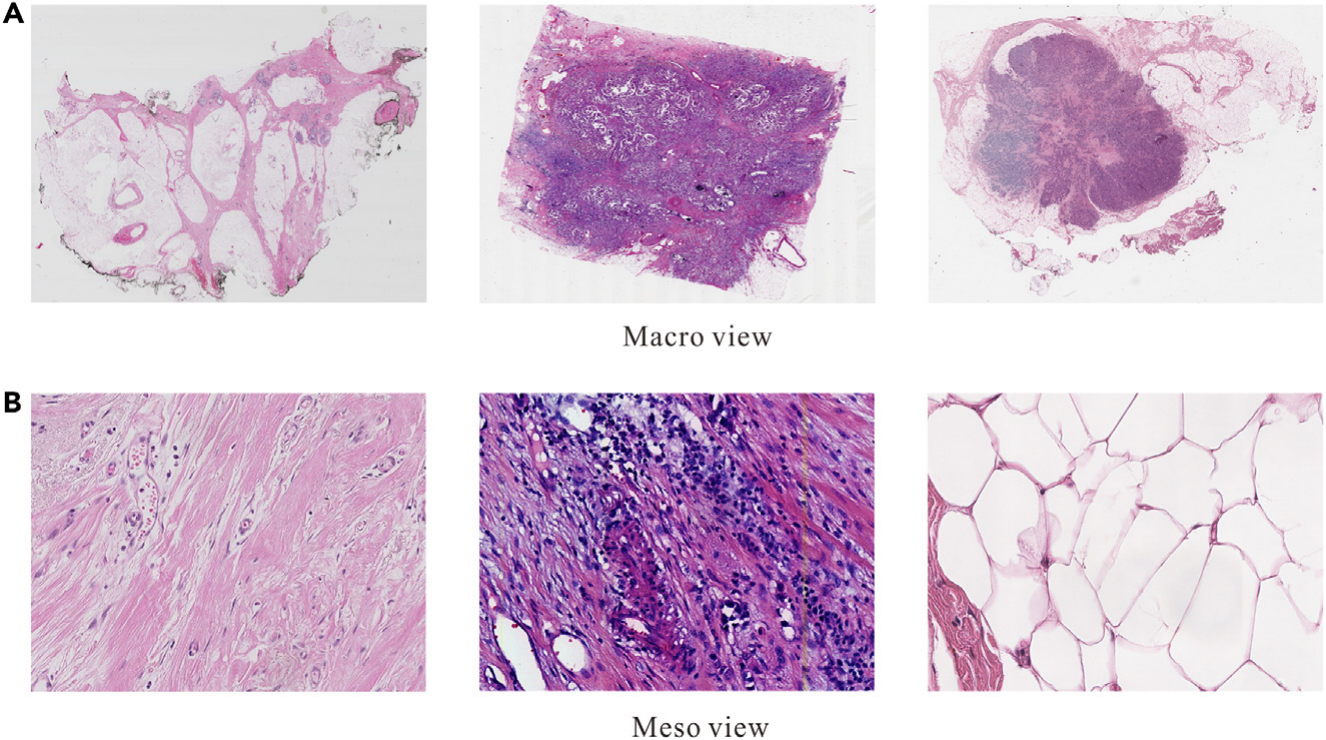
Strong heterogeneity of WSI from macro and meso perspectives

As depicted in Figure 12, WSIs exhibit strong heterogeneity and diversity. Various factors, such as different image acquisition devices and variations in the H&E staining process before scanning, can contribute to distribution drift in WSIs. Consequently, extracting nuanced prior knowledge from macro and meso perspectives becomes challenging.

**Figure 13:**
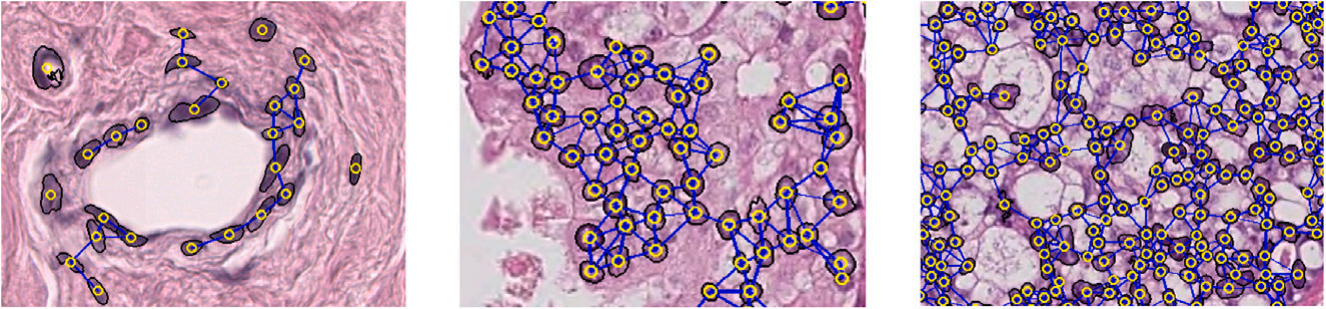
Nuclei in micro perspectives (the yellow circle is the nuclei centroid, the black circle is the nuclei contour, and the blue lines represent the adjacency relationship of nuclei)

As shown in Figure 13, the nucleus is a concrete object that can be quantitatively described. We can extract various attributes of the nucleus itself and analyze the interactions between nuclei. As indicated in Figure 10, the regions densely populated with nuclei coincide with areas of higher attention scores. Therefore, to enhance the interpretability of the model and broaden the dimension of feature analysis, it is necessary to extract multidimensional prior knowledge of nuclei and conduct quantitative analysis.

### How to incorporate prior knowledge into the AI model?

In the construction progress of deep learning modeling, the integration of prior knowledge can occur in three key phases: (1) During the initial design of the model structure, the relationship between differentiation and convolution can be harnessed to shape the convolutional kernel. The network structure can then be tailored using principles from the finite element method. (2) Handcrafted features can be introduced to the deep learning features at the bottom layer to augment feature dimensionality, followed by forward propagation layer by layer. The addition of tens of dimensions of handcrafted features to low-level deep learning features with thousands of dimensions may not necessarily enhance model performance. However, incorporating handcrafted features at the bottom layer becomes viable when the dimensionality of these features is sufficiently high. (3) At the top level of the model, the constraints of handcrafted features (prior knowledge) on the model are realized by adding penalty terms to the loss function. In this paper, we realize the constraints of handcrafted features on the classification results by adding a penalty term $L_{nuclei}$ to the model loss function.

### Conclusion

Recognizing the limitations of the current breast histopathology WSIs classification algorithm, which solely relies on slide-level and patch-level features while overlooking nuclei-level features, this paper introduces a NPKC-MIL for breast histopathology image classification. NPKC-MIL leverages the advantages of convolutional neural networks in model fitting while also imbuing the model with clear physical significance by the introduction of constraints based on handcrafted features. Experimental results demonstrate that, compared to state-of-the-art methods, the proposed approach exhibits superior specificity, sensitivity, accuracy, and precision in the classification of breast histopathology WSIs. This offers valuable insights for expanding the analytical scope of current WSI classification tasks and incorporating prior knowledge (handcrafted features) into deep learning models.

### Limitations of the study

While we confirmed the effectiveness of integrating prior knowledge into deep learning models, our approach was limited to employing significant 16-dimensional handcrafted features derived from the statistics of 1 million nuclei. Unfortunately, color features and contour features were not considered in NPKC-MIL. Moreover, our experiments were exclusively conducted on breast WSIs. To advance our understanding, future research should focus on exploring additional prior knowledge and conducting tests on diverse cancer types.

## STAR★Methods

### Key resources table

**Table:** 

REAGENT or RESOURCE	SOURCE	IDENTIFIER
**Deposited data**		

BRACS	BRACS	https://www.bracs.icar.cnr.it/
BACH2018	BACH2018	https://iciar2018-challenge.grand-challenge.org/Dataset/

**Software and algorithms**		

Convex hull	Sklansky,J.^47^ (1982)	https://doi.org/10.1016/0167–8655(82)90016-2
Hover-net	Graham et al.^48^ (2019)	https://doi.org/10.1016/j.media.2019.101563
K-NN	Cover et al.^49^ (1967)	https://doi.org/10.1109/TIT.1967.1053964
Graph convolutional	Kipf et al.^50^ (2017)	https://doi.org/10.48550/arXiv.1609.02907
Neighborhood aggregation	Corso et al.^51^ (2020)	https://doi.org/10.48550/arXiv.2004.05718
ResNet	He et al.^52^ (2016)	https://doi.org/10.1109/CVPR.2016.90
APL loss	Ma et al.^53^ (2020)	https://doi.org/10.48550/arXiv.2006.13554
Adam	Kingma et al.^54^ (2017)	https://doi.org/10.48550/arXiv.1412.6980
NPKC-MIL	This study	https://github.com/WxpHB/NPKC-MIL

### Resource availability

#### Lead contact

Further information and requests for resources should be directed to and will be fulfilled by the lead contact, Wei Yuan (e-mail: wei.yuan@tohoku.ac.jp).

#### Materials availability

This study did not generate new unique reagents.

#### Data and code availability

• BRACS dataset was downloaded https://www.bracs.icar.cnr.it/. BACH2018 dataset was downloaded from https://iciar2018-challenge.grand-challenge.org/Dataset/.
• All original code has been deposited at Github (https://github.com/WxpHB/NPKC-MIL) and is publicly available as of the date of publication.
• Any additional information required to reanalyze the data reported in this paper is available from the lead contact upon request.

### Method details

#### Principle of nuclei-level feature design

##### Geometric features

Mark any binary map of nuclei region in the image as $H_{n\times m}$, its (p+q)-order origin moment $M_{p,q}$ and central moment $\mu_{p,q}$ are:

(Equation 1) $$M_{p,q}=\sum_{x=0}^{n}\sum_{y=0}^{m}x^{p}y^{q}H_{n\times m}\left(x,y\right)$$

(Equation 2) $$\mu_{p,q}=\sum_{x=0}^{n}\sum_{y=0}^{m}{\left(x-\bar{x}\right)}^{p}{\left(y-\bar{y}\right)}^{q}H_{n\times m}\left(x,y\right)$$

where, $\bar{x}=\frac{M_{1,0}}{M_{0,0}}$, $\bar{y}=\frac{M_{0,1}}{M_{0,0}}$.

Then, the 9-dimensional geometric features can be calculated as follows:

(Equation 3) $$Major=\sqrt{8\left({\bar{\mu }}_{2,0}+{\bar{\mu }}_{0,2}+\sqrt{4{\bar{\mu }}_{1,1}^{2}+{\left({\bar{\mu }}_{2,0}-{\bar{\mu }}_{0,2}\right)}^{2}}\right)}$$

(Equation 4) $$Minor=\sqrt{8\left({\bar{\mu }}_{2,0}+{\bar{\mu }}_{0,2}-\sqrt{4{\bar{\mu }}_{1,1}^{2}+{\left({\bar{\mu }}_{2,0}-{\bar{\mu }}_{0,2}\right)}^{2}}\right)}$$

(Equation 5) $$Area=M_{0,0}=\sum_{x=0}^{n}\sum_{y=0}^{m}H_{n\times m}\left(x,y\right)$$

(Equation 6) $$\theta =\frac{1}{2}\arctan\left(\frac{2{\bar{\mu }}_{1,1}}{{\bar{\mu }}_{2,0}-{\bar{\mu }}_{0,2}}\right)$$

(Equation 7) $$Ecc=\sqrt{1-\frac{Minor^{2}}{Major^{2}}}$$

(Equation 8) $$Ell=\frac{Minor}{Major}$$

(Equation 9) $$Dia=\sqrt{4\frac{Area}{\pi }}$$

(Equation 10) $$Per=N_{v}+N_{h}+\sqrt{2}N_{d1}+\sqrt{2}N_{d2}$$

(Equation 11) $$AreaHull=\sum_{x}\sum_{y}T\left(x,y\right)$$

where, ${\bar{\mu }}_{2,0}=M_{2,0}/M_{0,0}-{\bar{x}}^{2}$, ${\bar{\mu }}_{0,2}=M_{0,2}/M_{0,0}-{\bar{y}}^{2}$, ${\bar{\mu }}_{1,1}=M_{1,1}/M_{0,0}-\bar{x}\;\bar{y}$, $N_{v}$, $N_{h}$, $N_{d1}$, and $N_{d2}$ represent the number of adjacent pixels of the pixels on the nuclear contour in the directions 0°, 90°, 45°, and 135°, respectively; T is the convex hull^47^ containing the nuclei.

##### Texture features

Let the GLCM (Gray Level Co-occurrence Matrix, GLCM) of $F_{n\times m}$ as $G_{l\times l}$ and meets:

(Equation 12) $$G_{l\times l}\left(i,j\right)=\sum_{x=1}^{n}\sum_{y=1}^{m}\left\{ \begin{array}{cc} 1, & if\;F_{n\times m}\left(x,y\right)=i\;and\;F_{n\times m}\left(x+\Delta x,y+\Delta y\right)=j;\\ 0, & \;else. \end{array}\right.$$

where, i and j are gray values; (Δx,Δy) is the offset of the pixel (x,y),. (Δx,Δy) takes values of 1,0, 1,1, 0,1, and −1,1, representing the commonly used four directions of 0°, 45°, 90°, and 135°, respectively, $F_{n\times m}$ is the grey-scale map of nuclei region in the image, l is the grayscale level in $F_{n\times m}$.

Then, 7-dimensional texture features can be outlined as follows:

(Equation 13) $$Con=\sum_{i=0}^{l-1}\sum_{j=0}^{l-1}{\left(i-j\right)}^{2}G\left(i,j\right)$$

(Equation 14) $$Diss=\sum_{i=0}^{l-1}\sum_{j=0}^{l-1}G\left(i,j\right)\left|i-j\right|$$

(Equation 15) $$\mathrm{Hom}=\sum_{i=0}^{l-1}\sum_{j=0}^{l-1}\frac{G\left(i,j\right)}{1+{\left(i-j\right)}^{2}}$$

(Equation 16) $$Ent=-\sum_{i=0}^{l-1}\sum_{j=0}^{l-1}G\left(i,j\right)\log\;G\left(i,j\right)$$

(Equation 17) $$ASM=\sum_{i=0}^{l-1}\sum_{j=0}^{l-1}G{\left(i,j\right)}^{2}$$

(Equation 18) $$Rou=\frac{P_{h}}{P_{n}}$$

(Equation 19) $$Dsip=\sqrt{\sum_{i=0}^{l-1}\sum_{j=0}^{l-1}{\left(G\left(i,j\right)-\epsilon \right)}^{2}}$$

where, $P_{h}$ and $P_{n}$ are the perimeter of the nuclei and its convex hull, respectively, ε is the mean of $G_{l\times l}$.

Subsequently, the handcrafted features are aggregated and updated following Equation 21. The optimization is performed iteratively using the Adam optimizer to refine the Equation 32.

#### Nuclei-level topology map construction and node message passing

Based on the fact that "the interaction of nuclei closer in space is stronger, while the interaction of nuclei farther away is weaker",^55^ Firstly, utilize the Hover-Net model^48^ to extract nuclei and abstract them into nodes, denoted as v. Then, employ the K-NN algorithm^49^ to construct an undirected graph of nodes, marked as G=(V,E), where V is a set of nodes and E is a set of edges. The principle of edge construction is as follows: $\forall v_{i}\in V$ when a node $\forall v_{j}\in V$ satisfies Equation 20, construct an undirected edge connecting $v_{i}$ and $v_{j}$, denoted as $e_{ij}=\left(v_{i},v_{j}\right)\in E$.

(Equation 20) $$v_{j}\in \left\{w|dist\left(v_{i},w\right)\leq d_{K}\wedge dist\left(v_{i},w\right)<d_{\min},\forall v_{i},w\in V\right\}$$

where dist(v,w) is the distance function, this paper takes Euclidean distance; $d_{K}$ is the nearest neighbor of K in dist(v,w); $d_{\min}$ is the nearest neighbor threshold. In this experiment, K takes 5 and $d_{\min}$ takes 50.

After constructing the node topology graph, extract 16-dimensional handcrafted features such as geometric and texture features of nodes according to the method described in the principle of nuclei-level feature design section. Finally, according to graph convolution theory^50^ and node message passing mechanism,^51^ node messages are aggregated and updated according to Equation 21.

(Equation 21) $$h^{\left(t+1\right)}\left(v\right)=U\left(h^{\left(t\right)}\left(v\right),\underset{u\in N\left(v\right)}{⊕}M\left(h^{\left(t\right)}\left(v\right),\underset{u\in N\left(v\right)}{h^{\left(t\right)}}\left(u\right)\right)\right)$$

where $h^{\left(t\right)}\left(v\right)$ and $h^{\left(t+1\right)}\left(v\right)$ are the feature vectors of the current layer and the next node layer v, respectively. $\underset{u\in N\left(v\right)}{h^{\left(t\right)}}\left(u\right)$ is the adjacent node feature vectors of the node v; U, M are multi-layer perceptron; N(v) is the adjacency matrix of the node v; ⊕ is a multi-scale scaling and aggregation operator that satisfies:

(Equation 22) $$⊕=\left[\begin{array}{c} I\\ S\left(D_{r\times r},\alpha =1\right)\\ S\left(D_{r\times r},\alpha =-1\right) \end{array}\right]⊗\left[\begin{array}{c} \mu \\ \sigma \\ \max\\ \min \end{array}\right]$$

where ⊗ is the tensor product, I is the identity matrix, $D_{r\times r}$ is the degree matrix of nodes, and r is the number of nodes in a patch; α is the scaling factor, shrinking is negative, magnifying is positive; μ, σ, max , min are the mean value, standard deviation, maximum value and minimum value of node features respectively; S is the degree scaling matrix and meets:

(Equation 23) $$S\left(D_{r\times r},\alpha \right)={\left(\frac{\log\left(D_{r\times r}+I\right)}{\delta }\right)}^{\alpha }$$

where δ is the normalized index calculated based on training data, and satisfy:

(Equation 24) $$\delta =\frac{1}{T_{n}}\sum_{i=0}^{r-1}\sum_{j=0}^{r-1}\log\left(D_{r\times r}\left(i,j\right)+1\right)$$

where $T_{n}$ is the number of nodes for each input of training data.

#### Patch-level feature extraction and slide-level feature construction

The ResNet 50 network model,^52^ pre-trained on ImageNet, serves as the patch-level feature extractor in our experiment. Following the third residual module of the network, we apply global average pooling to transform the input patch of dimensions 512 × 512 into a 1 × 1024 output feature vector, which is then aggregated into slide-level features. Initially, all patches are assigned weights based on the multi-head attention mechanism, as per Equation 25. Subsequently, we select c samples (c = 8) with the highest attention values as the training samples for the nuclei classifier. Finally, the attention pooling method is employed to aggregate patch-level features into slide-level characteristics, as indicated by Equation 26.

(Equation 25) $$s_{i}=\frac{\exp\left\{\tau \left(SA_{a}p_{i}^{⊤}\right)⊙\xi \left(SA_{b}p_{i}^{⊤}\right)\right\}}{\sum_{i=1}^{N}\exp\left\{\tau \left(SA_{a}p_{i}^{⊤}\right)⊙\xi \left(SA_{b}p_{i}^{⊤}\right)\right\}}$$

(Equation 26) $$f_{slide}=\sum_{i=1}^{N}s_{i}p_{i}$$

where $s_{i}$ is the weight of the i-th patch(i = 1,2, …, *N*); $p_{i}$ is features vector of the i-th patch; $f_{slide}$ is the slide-level features vector. $SA_{a}$, $SA_{b}$ indicates the multi-head attention, and meets:

(Equation 27) $$SA\left(Q,K,V\right)=\text{softmax}\left(\frac{QK^{⊤}}{\sqrt{d_{K}}}\right)V$$

(Equation 28) $$\text{softmax}\left(z\right)=e^{z_{i}}/\sum_{i=1}^{n}e^{z_{i}}$$

sofamx is the normalized function. $QK^{⊤}/\sqrt{d_{K}}$ is the self-attention matrix, performing linear transformations $K=W^{K}X$, $Q=W^{Q}X$, $V=W^{V}X$ on the input image X to obtain Q (Queries), K (Keys), V (Value); $W^{Q}$, $W^{K}$ and $W^{V}$ are the learnable transformation matrix; τ(x), ξ(x) are the activation function, and meets:

(Equation 29) $$\tau \left(x\right)=\frac{1}{1+e^{-x}};\;\xi \left(x\right)=\frac{e^{x}-e^{-x}}{e^{x}+e^{-x}}$$

#### Classify WSIs based on nuclei-level handcrafted feature constraint

The attention mechanism serves to identify potential ROI (Regions of Interest, ROIs). However, it’s important to note that a region with a high attention score may not necessarily indicate a diseased area, and conversely, a region with a low attention score may not always be normal. This implies that selecting Top-K ROIs based on attention scores introduces noise.^56^ To address this, this paper employs the noise-robust APL loss function^53^ for both patch-level and nuclei-level, as per Equation 30, and utilizes the cross-entropy error function from Equation 31 for slide-level losses. The slide-level losses are then constrained by incorporating patch-level and nuclei-level losses as penalty terms to formulate the optimization objective function in Equation 32. Finally, a WSI classification model is constructed by approximating the minimum value of Equation 32 using the Adam optimizer.^54^

(Equation 30) $$L_{patch,nuclei}=\log_{\prod_{k=1}^{K}p\left(k|x\right)}p\left(y|x\right)-\sum_{k=1}^{K}p\left(k|x\right)\log\left(q\left(k|x\right)\right)$$

(Equation 31) $$L_{slide}=-\sum_{i=1}^{K}q\left(k|x\right)\log\left(p\left(k|x\right)\right)$$

(Equation 32) $$L_{total}=w_{1}L_{slide}+w_{2}L_{patch}+w_{3}L_{nuclei}$$

where q(k|x) is the truth distribution; p(k|x) is the predicted value distribution; $L_{slide}$, $L_{patch}$, $L_{nuclei}$, and $L_{total}$ are slide-level, patch-level, nuclei-level, and total losses, respectively. In this paper, let $w_{1}$ = 0.7, $w_{2}$ = 0.2, $w_{3}$ = 0.1.

#### Evaluation criteria

The commonly used classification evaluation criteria are accuracy (AC), specificity (SP), which measures an algorithm’s ability to recognize normal WSIs, sensitivity (SE), also known as recall rate, which measures the power of the algorithm to identify cancerous WSIs, and precision (PC). Giving GT (Ground Truth) as the truth label, donate normal WSI as GT = 0 and cancerous one as GT = 1. Let PreCls (Predicted Class) be the predicted result of the algorithm, then:

(Equation 33) $$AC=\frac{TP+TN}{TP+TN+FP+FN};SP=\frac{TN}{TN+FP}$$

(Equation 34) $$SE=\frac{TP}{TP+FN};\;PC=\frac{TP}{TP+FP}$$

where TP (True Positive) means GT = 1 and PreCls = 1; TN (True Negative) means GT = 0 and prelude = 0; FN (False Negative) means GT = 1 and PreCls = 0; FP (False Positive) means GT = 0 and PreCls = 1.

### Quantification and statistical analysis

All statistical details, such as accuracy and sensitivity, can be located in the results, methods, and/or figure legends.
